# C_2_H_2_-Type Zinc Finger Proteins in Brain Development, Neurodevelopmental, and Other Neuropsychiatric Disorders: Systematic Literature-Based Analysis

**DOI:** 10.3389/fneur.2020.00032

**Published:** 2020-02-14

**Authors:** Njoud Al-Naama, Rafah Mackeh, Tomoshige Kino

**Affiliations:** Laboratory of Molecular and Genomic Endocrinology, Division of Translational Medicine, Sidra Medicine, Doha, Qatar

**Keywords:** brain development, structural abnormality, KRAB domain, mutation, neural stem cells, transcriptional regulation

## Abstract

Neurodevelopmental disorders (NDDs) are multifaceted pathologic conditions manifested with intellectual disability, autistic features, psychiatric problems, motor dysfunction, and/or genetic/chromosomal abnormalities. They are associated with skewed neurogenesis and brain development, in part through dysfunction of the neural stem cells (NSCs) where abnormal transcriptional regulation on key genes play significant roles. Recent accumulated evidence highlights C_2_H_2_-type zinc finger proteins (C_2_H_2_-ZNFs), the largest transcription factor family in humans, as important targets for the pathologic processes associated with NDDs. In this review, we identified their significant accumulation (74 C_2_H_2_-ZNFs: ~10% of all human member proteins) in brain physiology and pathology. Specifically, we discuss their physiologic contribution to brain development, particularly focusing on their actions in NSCs. We then explain their pathologic implications in various forms of NDDs, such as morphological brain abnormalities, intellectual disabilities, and psychiatric disorders. We found an important tendency that poly-ZNFs and KRAB-ZNFs tend to be involved in the diseases that compromise gross brain structure and human-specific higher-order functions, respectively. This may be consistent with their characteristic appearance in the course of species evolution and corresponding contribution to these brain activities.

## Introduction

Neurodevelopmental disorders (NDDs) are multifaceted pathologic conditions caused by skewed development of the central nervous system (CNS) and subsequent morphological and/or functional abnormalities (1). Manifestations associated with NDDs include, but are not limited to, neuropsychiatric problems, cognitive impairment, motor dysfunctions, language/speech abnormalities, and affective deficits (2). Intellectual disability (ID), autism spectrum disorders (ASDs), motor diseases including developmental coordination disorder, communication, speech and language disorders, attention-deficit/hyperactivity disorder (ADHD), and various genetic disorders, such as Down syndrome and fragile-X syndrome, all fall into the NDD entity (1). Neuropsychiatric disorders like schizophrenia, major depressive disorder (MDD), and bipolar affective disorder (BAD) are also considered as part of the NDDs (1). There are significantly overlapping clinical symptoms between different types of NDDs (3), suggesting the presence of commonalities shared among them. The pathologic mechanisms developing NDDs emerge during the early stage of brain development organized *in utero* and in childhood, and this is largely due to significant involvement of genome deficits in various key genes required for normal brain development (2). Thus, identifying causative mutations/genetic abnormalities greatly facilitates our understanding of the overall pathogenesis and neuropathological processes of NDDs.

One of the major requirements for normal brain development is the precise coordination of neural stem cell (NSC) activity throughout the embryonic period to early childhood (4). NSCs are self-renewing multipotent cells that give rise to three distinct types of CNS cells: neurons, astrocytes, and oligodendrocytes (4). Differentiated neurons are critical for virtually all brain activities including the coordination of sensory and motor systems, cognitive functions, and mood maintenance (5). On the other hand, astrocytes and oligodendrocytes, also known as glial cells, support proper functioning of the differentiated neurons (5). During the early stage of embryonic brain development, NSCs originate from the neuroepithelial stem cells of the embryonic neural tube (6). NSCs undergo three major stages: (1) proliferation and renewal of the lineage, (2) migration to appropriate brain areas, and (3) differentiation into neurons, astrocytes, or oligodendrocytes; precise transitioning between these stages is critical for normal brain development (7). For example, transitioning from proliferation to differentiation and subsequent induction of the programmed cell death are crucial for the formation of normal anatomical structure of the developing brain by maintaining appropriate cell numbers (8, 9). Importantly, many of these NSCs activities are orchestrated and driven by the spatio-temporal expression of the groups of genes responsible for fine-tuning of transcriptional activity (10). Thus, dysregulation in any processes supported by these key genes impacts proper NSC activities, resulting in the development of NDDs (11, 12).

Transcription factors (TFs) are a family of protein molecules that drive gene transcription by binding directly/indirectly to the upstream genome regulatory elements of protein-coding genes (13). Accumulated evidence indicates that TFs are pivotal for brain development by influencing the ability of NSCs to differentiate into different neural cell lineages and the subsequent formation of various brain areas and substructures (14). Some TFs are also key for the precise neural cell migration to their final brain destinations (14, 15). Among such TFs, the C_2_H_2_-type zinc finger proteins (C_2_H_2_-ZNFs) are highlighted to play significant roles in the regulation of NSCs activities and subsequent brain development (16–19). Many of their family members also participate in the pathogenesis and pathophysiology of NDDs (20). In this article, we will discuss the biological activities of C_2_H_2_-ZNFs in brain development and their pathologic contribution to NDDs.

## C_2_H_2_-ZNFs

**Figure 1:**
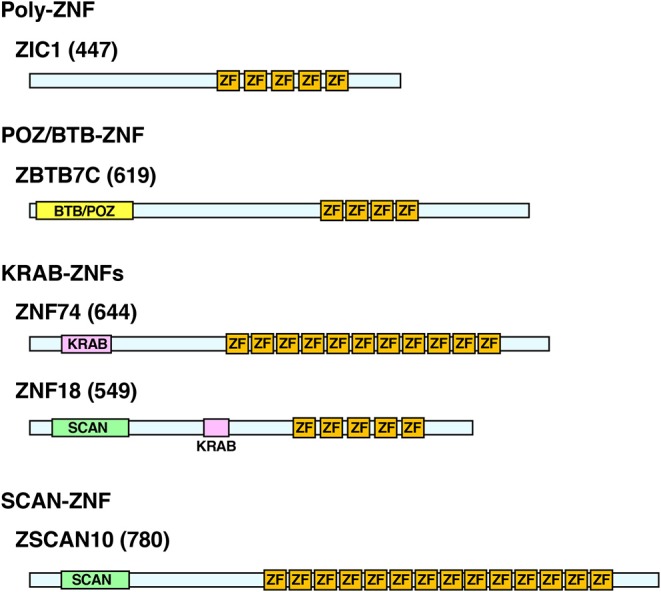
Protein structure of the representative human C_2_H_2_-ZNFs. Protein structure of ZIC1 (Poly-ZF), ZBTB7C (POZ/BTB-ZNF), ZNF74, ZNF18 (KRAB-ZNFs), and ZSCAN10 (SCAN-ZNF) are shown as representatives of respective subtypes. Some C_2_H_2_-ZNFs have multiples of the same or different domains as indicated in ZNF18. Location and length of the respective domains are based on the data from the UniProt (https://www.uniprot.org/) and/or the National Center of Biotechnology Information (NCBI) Conserved Domain (https://www.ncbi.nlm.nih.gov/Structure/cdd/wrpsb.cgi). Numbers in brackets indicate amino acid numbers. ZF, C_2_H_2_-type zinc finger; KRAB, KRAB domain; POZ/BTB, POZ/BTB domain; SCAN, SCAN domain.

C_2_H_2_-ZNFs form the largest TF family in the animal kingdom with significant expansion of their members through species evolution (20). The family consists of ~800 members in humans (20, 21). In addition to C_2_H_2_-type zinc fingers (ZFs), these proteins contain other functional domains, such as BTB (BR-C, ttk, and bab)/POZ (Pox virus and Zinc finger), KRAB (Krüppel-associated box), and/or SCAN (SRE-ZBP, CTfin51, AW-1, and Number 18 cDNA), and are classified into four subtypes depending on the possession of these domains: (1) poly-ZNFs without any other domains, (2) BTB/POZ-ZNFs, (3) KRAB-ZNFs, and (4) SCAN-ZNFs (20, 21) (Figure 1).

ZFs are small peptide domains forming a secondary structure supported by a zinc ion, which makes ionic bonds to the cysteine and/or histidine residues of the finger (22). The C_2_H_2_-type ZF is composed of up to 30 amino acids with the consensus sequence CX_2−4_CX_12_HX_2−8_H (X refers to any amino acid), which forms one α-helix and two β-sheets, respectively, in the carboxyl- and amino-terminal portions (23–25). These secondary structures of the C_2_H_2_-type ZF fold into a stable three-dimensional assembly through hydrophobic interactions and enclosure of a zinc ion (26, 27). In C_2_H_2_-ZNFs, multiple ZFs are usually present in tandem and are connected by linkers with conserved amino acid sequences (28). C_2_H_2_-type ZNFs bind genome DNA at their cognate recognition sequences located in the regulatory region of their target protein-coding genes by forming various modes of contacts to target DNA double helices with their ZFs. DNA-bound C_2_H_2_-type ZNFs then recruit cofactors, chromatin remodeling proteins and the RNA polymerase II together with other TFs, and modulate the transcription rates of downstream coding sequences (29). In addition to the primary role as DNA-binding factors, some C_2_H_2_-type ZNFs use their ZFs for interacting with other proteins or double-stranded RNAs, which may be important for their communication with other proteins/RNAs also attracted to the multi-molecule transcriptional complex formed on DNA (28, 30, 31).

Among the functional domains of C_2_H_2_-type ZNFs, BTB/POZ, and KRAB domains have transcriptional regulatory activity (mainly repressive but sometimes enhancing) by attracting various repressive cofactor molecules, such as the histone deacetylases, corepressor complex, heterochromatin protein 1 (HP1), and/or the KRAB-associated protein-1 (KAP1). In contrast, the SCAN domain does not have such activities (21, 32). About seven percent (%) of the human C_2_H_2_-type ZNFs have a BTB/POZ domain, while 43% harbor a KRAB domain and 7% contain a SCAN domain (33). Sixty-seven percent of them have only ZFs without any of these domains (33). Some C_2_H_2_-type ZNFs have multiples of the same or of different domains (29) (Figure 1).

C_2_H_2_-ZNFs are highly expressed in the developing brain, and control early patterning of the CNS (16). They significantly contribute to the regulation of brain morphogenesis, influencing the proliferation, migration, and cell fate of NSCs or one-step committed neural progenitor cells (NPCs), and their differentiation into neuronal cells (see below). Their implications to brain disorders still remain elusive, but recent clinical studies have identified various mutations in the coding sequences of many *C*_2_*H*_2_*-ZNF* genes in patients with NDDs (20). Hence, we will first discuss the physiologic roles of C_2_H_2_-ZNFs in normal brain development by focusing on their involvement in the actions of NSCs or NPCs (Table 1). We will then describe their involvement in the formation of some structural components of the CNS by introducing experimental and clinical evidence. Further, we will discuss their pathologic contribution to particular forms of NDDs.

**Table 1:** The C_2_H_2_-ZNFs involved in brain development, NDDs, and/or other neuropsychiatric disorders.

**Name**	**Additional domains**	**Biologic activities**	**Pathologic implications**	**References**
**Poly-ZNFs**				
ADNP2	Homeobox	Expressed in oligodendrocytes	Schizophrenia	(34, 35)
BCL11A (ZNF858A)		Controls migration of cortical neurons	ID, ASDs, seizures, dyspraxia, childhood apraxia of speech, severe speech disorder, brain malformation, and microcephaly	(17, 36, 37)
BCL11B (ZNF858B)		Controls hippocampal neurogenesis and development of corpus striatum		(38)
FEZF1 and FEZF2		Involved in cortical development and promote differentiation of neural stem cells Specifically expressed in subcortical projection neurons and regulates cell fate of residing neurons	Autism spectrum disorders and intellectual disabilities	(39–41)
GLI3		Controls progression of cell cycle in RGS cells through regulating the G1 phase length	Greig cephalopolysyndactyly syndrome, acrocallosal syndrome and Pallister-Hall syndrome	(9, 42, 43)
GLIS1		Promotes generation of the induced pluripotent stem cells	ASDs and Parkinson disease	(44, 45)
GLIS2		Regulates neuronal differentiation		(46)
TSHZ3	Homeobox	Influences synapse development by impairing cortico-striatal connectivity	Autistic traits, intellectual disabilities and speech disturbances	(47)
ZIC1		Controls cerebellar size	Dany-Walker malformation	(18)
ZIC2		Regulates migration of forebrain neurons, CR cells, and pallial-derived neurons	Holoprosencephaly and schizophrenia	(48–50)
ZIC3		Participates in neural crest formation, neurulation, and maintenance of NPCs	Hydrocephalus	(51–53)
ZIC4		Controls cerebellar size	Dany-Walker malformation	(18)
ZIC5		Mediates neural crest development and formation of the neural tube		(54)
ZNF148		Crucial for the development of corpus callosum	Underdevelopment of corpus callosum and aberrant neuron proliferation, microcephaly, and intellectual disabilities	(19)
ZNF292	Coiled coil		ID and ASDs	(55, 56)
ZNF385B		Mediates neuronal apoptosis	ASDs and ID	(57)
ZNF407			ID, ASDs and cognitive impairment.	(58)
ZNF462		Expressed in the ventricular zone and hippocampus Essential for hippocampal formation	ASDs	(59)
ZNF507			Schizophrenia	(60)
ZNF521		Promotes early neuronal differentiation	Anxiety and schizophrenic behavior	(61, 62)
ZNF536		Highly expressed in the developing CNS Promotes neural differentiation	MDD and BD	(63)
ZNF711	Zfx/Zfy transcription activation region	Activates the genes essential for brain development	ID	(64, 65)
ZNF774			ASDs	(66)
ZNF804A^*^		Implicates in brain connectivity (in the hippocampus and the dorsolateral prefrontal cortex) Implicates in episodic and working memory	Schizophrenia, ID, and ASDs	(67, 68)
ZNF865			ID and cerebral ataxia	(69)
**POZ/BTB-ZNFs**				
ZBTB7C		Highly expressed in the granular layers of the dentate gyrus and the pyramidal layer of the hippocampal gyrus	ID	(70)
ZBTB16			ASDs	(71)
ZBTB20		Highly expressed in the forebrain Involved in hippocampal neurogenesis, neuronal differentiation and neuronal connectivity Promotes astrocytogenesis	Macrocephaly (autistic features) Intellectual disabilities and autism	(72)
ZBTB21			Down syndrome	(73)
ZBTB32			MDD	(74)
ZBTB45		Highly expressed in the developing brain Regulates differentiation of glial progenitor cells, glial cells and oligodendrocyte precursors		(75)
ZmC_2_H_2_-1			Stress intolerance	(76)
**KRAB-ZNFs**				
PRDM15 (ZNF298)	SET	Acts in neural cell fate decision	Down syndrome and BD	(77, 78)
ZBTB11	Integrase H2C2		ID	(79)
ZBTB18		Coordinates corticogenesis and promotes radial cell migration	ID, microcephaly and corpus callosum anomalies.	(80)
ZEB1	Homeobox	Controls neuron differentiation Maintains integrity of the blood brain barrier	Schizophrenia	(81, 82)
ZEB2	Homeobox	Regulates the transition of radial glia to Bergmann glia	Mowar-Wilson syndrome	(83)
ZKSCAN4	SCAN		Schizophrenia	(67)
ZNF8		Transcriptional regulation	ASDs	(84)
ZNF18	SCAN	Regulates neuronal activity and/or development	Congenital form of ASDs	(85)
ZNF30			Microcephaly, intellectual disabilities, and poor speech development	(86)
ZNF34			MDD	(87)
ZNF41			XLMR and cognitive defects	(88)
ZNF74		Regulates of synaptic transmission	Schizophrenia and intellectual disabilities	(89, 90)
ZNF81			XLMR and autistic symptoms	(91)
ZNF181			Microcephaly, intellectual disabilities, and poor speech development	(86)
ZNF182			XLMR and autistic symptoms	(91)
ZNF302			Developmental delay, microcephaly, and intellectual disabilities	(86)
ZNF354C		Regulates gene expression during early embryonic brain development	Schizophrenia and depression	(92, 93)
ZNF439			Amyotrophic lateral sclerosis	
ZNF496	SCAN	Upregulated during the differentiation of P19 neural precursor cells	Epilepsy and hyperactivity Microcephaly and abnormal corpus callosum	(94, 95)
ZNF517			ASDs	(96)
ZNF519			Microcephaly, lissencephaly, and ID	(97)
ZNF528			ID	(98)
ZNF534			Epilepsy and ID	(99)
ZNF541			ID	(100)
ZNF546			ID	(101)
ZNF559			ASDs	(102, 103)
ZNF568		Maintains neuron stem cells and regulate neurogenesis	Microcephaly	(104)
ZNF589			ID	(105, 106)
ZNF599			Microcephaly, ID, and poor speech development	(86, 107)
ZNF673			ID and learning disabilities	(108)
ZNF674			ID and the X-linked cognitive disabilities	(108, 109)
ZNF713			ASDs and frontotemporal dementia	(110)
ZNF717			ID and polymicrogyria	(101, 111)
ZNF746	Coiled coil	Regulates neuronal death	Parkinson disease	
ZNF778			ASDs and cognitive impairment	(112)
ZNF780B			ID	(101)
ZNF860			Schizophrenia	(113)
ZNF862	HATC-C, RNase H-like		ID, language development, and information processing	(114)
**SCAN-ZNFs**				
ZNF24/ZNF191		Controls the transition stage from proliferation to differentiation in NPCs		(115)
ZSCAN10		Controls pluripotency of embryonic stem cells	Schizophrenia	(92)
ZSCAN31 (ZNF323)		Involves in early stages of brain development	Schizophrenia	(116)

* *ZNF804A has only one ZF*.

## Roles of C_2_H_2_-ZNFs in Normal Brain Development and Their Involvement in Brain Morphological Abnormalities

Embryonic brain development or morphogenesis begins with neurulation, the invagination of the neural plate to form the neural tube (4). Upon closure of the neural tube, the neuroepithelial cells residing in the ventricular zone shift from proliferative to neurogenic, and are committed into the radial glial progenitor cells (RGCs), which serve as the primary NPCs for generating neurons and glial cells (8). Neocortical development relies on different NPCs depending on their localization, such as apical progenitors (APs) and basal progenitors (BPs: also known as intermediate progenitors: IPs), which are, respectively localized in the apical surface and the basal side of the ventricular zone (117). Neurogenesis starts at E9-E13 in the mouse embryo in which RGCs go into two modes of cell division: “symmetric” to produce two daughter cells that retain the properties of RGCs, and “asymmetric” dividing into one daughter cell with the property of RGCs and one differentiated neural cell (8). The transition from symmetric to asymmetric division of RGCs is extremely critical for determining the numbers of residing neurons and subsequent brain size, whereas intrinsically coordinated cell cycle progression in these cells plays a role in balancing their proliferating *vs*. differentiating properties (9). Disruption of these processes thus leads to abnormal brain development (9).

Various C_2_H_2_-ZNFs are significantly involved in the above indicated process of neurogenesis organized by RGCs. The birth of cortical neurons is severely reduced or lost in *Gli3*-mutated mice (9). Gli3 is a poly-ZNF functioning in the sonic hedgehog (Shh) signaling and controls the cell cycle of RGCs by changing the length of the G1 phase (9, 42). Inactivation of Gli3 shortens the length of their entire cell cycle and causes delays in the formation of cortical neurons and the process of cortical lamination (9, 42). Several mutations in the *GLI3* gene are reported in patients with Greig cephalopolysyndactyly syndrome, Acrocallosal syndrome, and Pallister-Hall syndrome, which develop various morphological abnormalities in CNS and polydactyly (43).

The Zeb family of C_2_H_2_-ZNFs (Zeb1 and Zeb2), which are poly-ZNFs with one atypical homeodomain, is essential for normal brain development. Among them, Zeb1 is required for neocortical development (81). Its peak expression reaches during the period of neocortical development, persists at high levels throughout the embryonic neurogenesis and then decreases postnatally (81). Zeb1 acts as a transcriptional repressor and regulates proliferation, migration, and differentiation of RGCs by affecting the division mode of these cells (81). It promotes and accelerates maturation of the generated neurons and their ability to develop electrophysiological properties (81). Interestingly, inactivation of Zeb1 significantly decreases trans-differentiation from mouse embryonic fibroblasts into functional neurons in an *in vitro* system (81). Zeb2 (also known as Smadip1, Aip1, and Zfhxib) is essential for the transition of RGCs to Bergmann glia cells and astrocytes in mouse cerebellum (118). In humans, the *ZEB2* mutation is associated with Mowat-Wilson syndrome, a genetic disorder characterized by ID, epilepsy, and motor defects (119–121).

The Zic-type poly-ZNFs (Zic1, Zic2, Zic3, Zic4, and Zic5) are expressed in the specific regions of neuroectoderm during the early embryonic phase in mice, and they have essential roles in CNS development (18, 51, 54). Specifically, Zics are pivotal for regulating the proliferation and the differentiation of NPCs in the medial forebrain and cerebellum (122), and are involved in the neurulation process and neural tissue formation (63). They are essential for the neural tube formation, particularly the neural plate closure (122). Zics expressed in the neural tube seem to play a role in the formation of the neural crests as well (122). They also contribute significantly in forebrain development, as mutations in *Zic1, Zic2*, and *Zic3* result in an inadequate division of forebrain, which fails to develop into two hemispheres (123). Indeed, Zic1, Zic2, and Zic3 are expressed in the NPCs residing in the septum and cortical hem, the sites of generation of the Cajal-Retzius (CR) cells. Mice defective in these Zics demonstrate a reduction in the number of CR cells in the rostral cortex and develop altered localization of the CR cells and cortical lamination defects that resemble the changes noted in type II (cobblestone) lissencephaly (124). Zic1 and Zic4 are also involved in cerebellar morphogenesis (123). Simultaneous deletion of the *ZIC1* and *ZIC4* genes due to their close proximity in chromosome 3 results in a congenital brain anomaly called Dany-Walker malformation (DWM) in humans, which is characterized by hypoplasia of the cerebellar vermis and other brain abnormalities, and develops delayed motor development and cognitive problems in the affected individuals (18).

Several C_2_H_2_-ZNFs are implicated in the etiology of microcephaly as well. Microcephaly refers to a reduction in brain circumference and diminution in brain volume (125). The majority of cases with microcephaly are congenital forms in which the processes of neuronal proliferation, migration and/or death are affected (125). *De novo* deletions in the 19q13.11 region encompassing four *KRAB-ZNFs* (*ZNF30, ZNF81, ZNF302*, and *ZNF599*) are identified in two unrelated cases of microcephaly (86). Both patients demonstrated mild to severe ID and speech disturbances (86) and, in mice, microcephaly developed when the *Znf568* gene was knocked out (104). Znf568 is the KRAB-ZNF essential for NSC maintenance and brain size regulation (104). Znf568 is expressed in NSCs of fetal mouse brain (104). It is also expressed in the adult NSCs residing in two neurogenic niches, the subgranular zone (SGZ) and the subventricular zone (SVZ) of the hippocampal dentate gyrus (104). Mice defective in *Znf568* develop a significantly smaller brain compared to wild type mice (104). Reduction of the brain size in these mice is mainly due to defective neuronal migration and subsequent abnormal cortical layering (104). Further, particular single nucleotide variants in the *ZNF568* gene are associated with the head size in humans (104).

ZNF519 is a poly-ZNF highly expressed in brain and is involved in the etiology of microcephaly and lissencephaly (126). The latter is a developmental malformation of the brain cortex (smooth brain without normal convolutions) caused by improper neuronal migration (126). Investigation on a four-generation Pakistani consanguineous family exhibiting congenital microcephaly (Jawad syndrome) and remarkable learning deficits mapped the causative gene(s) to the chromosome 18p11.22-q11.2, which harbors six candidate genes including *ZNF519* (97). The potential contribution of ZNF519 to the development of lissencephaly is also supported by the evidence that its expression is downregulated in mice with *Lis1, Dcx*, or *Ywhae* knockouts, whose gene mutations are causative for lissencephaly in humans (126). Zfp462, a poly-ZNF involved in the pluripotency and differentiation of embryonic stem cells by regulating the expression of Sox2, Oct4, and Nanog TFs in mice (127), modulates the expression of the genes specific to neuronal differentiation (59). It is predominantly expressed in the embryonic cerebral cortex particularly in the ventricular zone and hippocampus (59). Homozygotic *Zfp462* knockout is lethal in mice, whereas the heterozygotic mice exhibit developmental delay with low brain weight and anxiety-like behavior with excessive self-grooming (59). ZNF148 is a poly-ZNF associated with congenital brain structural defects in humans. ZNF148 is highly expressed in the developing fetal brain in humans and is crucial for the development of the corpus callosum (19). Four patients harboring *de novo* truncating mutations in the *ZNF148* gene shared core syndromic features including abnormal development of corpus callosum, microcephaly, ID, short stature, and facial dimorphisms (19).

ZNF521 is the KRAB-ZNF acting as one of the intrinsic factors for driving commitment of NSCs to NPCs (61). It also promotes proliferation of these cells and delays their differentiation (61). ZNF24/ZNF191 is a KRAB-ZNF also harboring one SCAN domain (128). ZNF24/ZNF191 is expressed in NPCs, and is required for the maintenance of their proliferation potency by promoting cell cycle progression (115). Accordingly, ZNF24/ZNF191 expression is pronounced during early brain development and its expression decreases after all differentiation occurs (115).

## Involvement of C_2_H_2_-ZNFs in ID

ID is one type of the generalized NDDs characterized by significant impairment of intellectual (such as learning and reasoning) and adaptive functioning (activities for daily living, such as communication and independent living) (129). It is a heterogeneous disorder with regard to its clinical and genetic characteristics (130). Some C_2_H_2_-ZNFs are involved in the development of ID, particularly the form called X-linked intellectual disability (XLID) (131). This genetic disease is inherited in an X-linked repressive fashion, and thus, affected boys demonstrate more obvious phenotypes than girls (131). Several *KRAB-ZNF* genes, such as *ZNF41, ZNF81, ZNF148, ZNF673*, and *ZNF674*, residing on chromosome X are reported as novel causative genes for XLID with strong association to particular phenotypes among the other ~200 candidate genes (109, 132). Several unrelated ID patients displaying similar manifestations and developmental delays shared the same mutations in the *ZNF674* and *ZNF673* genes (108, 109). Four patients harboring *de novo* truncation mutations in the *ZNF148* gene demonstrated overlapping clinical manifestations including ID, microcephaly, and mal-development of the corpus callosum (19). Two mutations in the *ZNF711* gene, which is also located on chromosome X and encodes a poly-ZNF protein, were identified in 11 XLID patients from two families, some of whom additionally demonstrated autistic features (64). ZNF711 has a role in brain development by binding to the PHD finger protein 8 (PHF8) that is the histone demethylase highly expressed in neurons, and failure of ZNF711 to bind to PHF8 affects normal neuronal migration (64, 65). Mutations in the *PHF8* gene, which is also located on chromosome X, cause Siderius-type XLID, characterized by facial dysmorphism, cleft lip/palate, and occasionally microcephaly and ID (133, 134).

## Involvement of C_2_H_2_-ZNFs in ASDs and Down Syndrome

ASDs are a group of pervasive NDDs demonstrating heterogeneous manifestations mainly characterized by deficits in social cognition, communication, and restricted behavior with repetitive phenotypes (135). They can range from mild social cognitive impairment to debilitating cognitive abilities (135). Accumulating evidence indicates the significant contribution of C_2_H_2_-ZNFs in the pathogenesis and pathophysiology of ASDs and autistic features. These include BCLLA, FEZF1, FEZF2, GLIS1, POGZ, TSHZ3, ZBTB16, ZBTB20, ZNF8, ZNF18, ZNF81, ZNF182, ZNF292, ZNF385B, ZNF407, ZNF462, ZNF517, ZNF548, ZNF559, ZNF626, ZNF713, ZNF774, ZNF778, ZNF804A, ZNF827, and many of them are KRAB-ZNFs (Table 1). Below, we explain the contributions of some of these C_2_H_2_-ZNFs in the development of ASDs and autistic features.

A 335.4 Kb-size microduplication located in the Xp11.2p11.3 segment of chromosome X, which includes KRAB-ZNF-expressing *ZNF81* and *ZNF182*, was identified in a patient demonstrating developmental retardation, autistic features, and delayed growth and speech (91). Elevated ZNF182 expression was identified in another ASD case displaying hyperactivity, learning and visual-spatial difficulties, and microcephaly (136). The latter patient harbored a 1.3 Mb-size micro-duplication in Xp11.23p11.3 that includes *ZNF182* (136). These two cases suggest that elevated expression of ZNF182 with the dosage nature of its encoding gene contributes to the development of their ASD phenotypes. *ZNF292* is also a potential target gene for ASDs (55, 56). One study employing a large cohort of the ASD probands obtained from the Autism Clinical and Genetic Resourced in China (ACGC) indicated *ZNF292* as a novel autism risk gene, as the patients harboring various mutations in this gene demonstrated ID and severe language impairment (55). Another study using a large ASD cohort collected from several countries identified four unrelated individuals who had deletions of the *ZNF292* gene (56). Homozygotic and compound heterozygotic mutations in the *ZNF18* gene were identified in the Autism Genetic Research Exchange (AGRE) cohort consisting of ~1,000 multiplex ASD families (85). ZNF18 is a KRAB-ZNF with one SCAN domain, and is upregulated upon depolarization in mouse neuronal cells, suggesting its potential activity-dependent roles (85). The *ZBTB20* gene is also involved in the development of ASDs in addition to other types of NDDs including 3q13.31 microdeletion and microduplication syndrome, Primrose syndrome and ID (72, 137–139). ZBTB20 is a BTB/POZ-ZNF mainly expressed in the developing forebrain neocortex and is involved in cortical neurogenesis, hippocampal neuronal differentiation and connectivity, and promotes astrocytogenesis (140). Four unrelated individuals with *de novo* inactivating mutations in the Krüppel-like factor 7 (*KLF7*) gene exhibited autistic features along with ID (141). KLF7 is a poly-ZNF, and is essential for neurogenesis and is involved in neuronal differentiation and morphogenesis (141, 142). *Klf7*-knockout mice showed impaired axon projection in several brain regions including the cerebral cortex and hippocampus, and exhibited reduced dendritic branching in hippocampus (142). The pogo transposable element with ZNF domain (*POGZ*) gene is also a plausible candidate for ASDs, as *de novo* missense or nonsense mutations in this gene were identified in at least eight independent ASD patients (143–145). POGZ, a unique poly-ZNF harboring the transposase domain at its C-terminus in addition to nine ZFs (145), is highly expressed in the human fetal brain and is involved in mitosis and regulation of neural proliferation (145). POGZ is also implicated in the development of NDDs and microcephaly, as several *de novo* loss-of-function mutations in this gene were identified in seven patients showing these manifestations (146).

Several C_2_H_2_-ZNFs have etiologic roles in the manifestations associated with Down syndrome. Down syndrome is a common chromosomal disease caused by the chromosome 21 trisomy or its various rearrangements, and develops ID and constellations of morphological abnormalities (147). Some patients also demonstrate the manifestations reminiscent of ASDs (148). The Tc1 mouse model of Down syndrome shows elevated expression of Znf295 (also known as Zbtb21) in the brain cortex, and its human ortholog is located on chromosome 21, thus dosage abnormality in this BTB/POZ-ZNF may contribute to the development of some neurological manifestations associated with Down syndrome (149). Since *ZNF298* is located on chromosome 21q22.3 and duplication of this segment is strongly associated with the development of Down syndrome (150, 151), dosage abnormality in *ZNF298* appears to contribute to the development of some manifestations of this disease (152). ZNF298 is a poly-ZNF and has a SET [Su(var)3-9, Enhancer-of-zeste, Trithorax] domain in its N-terminal portion (152).

## Involvement of C_2_H_2_-ZNFs in Neuropsychitaric Diseases Including Schizophrenia, MDD, and BAD

Schizophrenia is a complex NDD characterized by psychotic symptoms, such as hallucinations and delusions, accompanied by variable degrees of loss of insight (153). Interplay between genetic, biological, environmental, and psychological factors are supposed to play roles in the development of these manifestations (89, 153). *ZNF74* was identified as a candidate gene for modifying the development of schizophrenia in particular patient groups (89). *ZNF74* encodes a KRAB-ZNF, is highly expressed in the developing brain and is located on chromosome 22q11, a gene segment previously identified as a positional candidate locus for the susceptibility to schizophrenia as part of the 22q11 deletion syndrome (154). ZNF74 is highly expressed in the developing fetal brain in humans (19). Several polymorphisms identified in *ZNF74* are significantly associated with age-at-onset of schizophrenia, although no statistical difference was detected for their frequencies between the patients and control subjects (89). Systematic meta-analysis on the psychotic diseases including schizophrenia, BAD, and ADHD identified several gene variants in *ZNF804A*, the zinc finger DHHC-type-containing 8 (*ZDHHC8*) and the zinc finger with KRAB and SCAN domain 4 (*ZKSCAN4*) genes (67). The *ZNF804A* variants, particularly rs1344706, located in the intronic sequence of this gene are highly associated with the development of schizophrenia and its various manifestations (155). *ZNF804A* expresses a poly-ZNF harboring just one ZF, and its reduced expression is likely important for the development of schizophrenia in part by changing the expression of the genes involved in neural cell adhesion, neurite outgrowth, and synapse formation (155). Although *ZDHHC8* is located on chromosome 22q11 and was initially identified as a potential candidate gene for schizophrenia, it turned out not to be involved in this disease in later studies (156, 157). The *ZKSCAN4* gene, also known as *ZNF307* or *ZNF427*, expresses a KRAB-ZNF that harbors a SCAN domain in its amino-terminus (158). This gene is located on chromosome 6p21p22.1, which was previously identified as one of the schizophrenia-associated gene loci (159). Several *ZKSCAN4* polymorphisms were strongly associated with schizophrenia in the Chinese Han population (160), although underlying molecular mechanisms are not known.

Mood disorders, such as MDD and BAD, are among the most common brain disorders caused by various abnormalities in the brain (e.g., imbalance of neurotransmitters), and particular genetic backgrounds precipitate these diseases (161). Several C_2_H_2_-ZNFs are involved in their pathogenesis. A novel point mutation in the *ZNF34* gene that replaces proline at the amino acid position 17 to arginine (P17R) was identified in a multi-generationally affected family with early-onset MDD (87). The mutation P17R is located in the KRAB-A domain of ZNF34, which is required for the repressive transcriptional activity of this protein, suggesting defective transcriptional regulation by the mutant protein appears to contribute to the development of MDD. *ZNF34* is also associated with BAD; *ZNF34* mRNA was differentially expressed in the postmortem brain samples obtained from patients with BAD (162). *ZNF34* also contains common variants precipitated in this disease (163). Further, *ZNF34* is located on chromosome 8p24.3, which is included in the region shown to be associated with BAD (164, 165). One *ZNF536* polymorphism (rs77554113) is correlated with remission rates of MDD patients who are under anti-depressant treatment, indicating its potential roles in MDD-related pathophysiologic processes (166). ZNF536 is a poly-ZNF highly expressed in neuronal cells and known to suppress neuronal differentiation (21, 63).

## Discussion

Brain development is organized by the sophisticated coordination of the proliferation, differentiation, migration, and cell death of its component neural cells (4). This is accomplished, mainly, by the intrinsic program of the self-renewing cell lineages, NSCs, and NPCs, through coordinated regulation of their transcriptional network by numerous TFs and transcriptional regulatory molecules (4). Importantly, these processes are under the influence of the individual's genetic background as well as the vulnerability to extrinsic factors, such as infectious agents, toxic substances, and various maternal conditions including immunity (2). Skewing any part of this regulatory network causes NDDs, leading to the development of various degrees of social, emotional, cognitive, and motor deficits (2).

Our literature-based analysis on the brain development and NDDs revealed that numerous C_2_H_2_-ZNF proteins (74, ~10% of all human member proteins) are essential or involved in these conditions (Table 1). Indeed, many of them play critical roles in the proper functioning of NSCs, such as their potencies of proliferation and commitment into differentiated neural cell lineages. We found that different C_2_H_2_-ZNFs act on specific functions of these self-renewing cells at the particular developmental stages and their residing brain areas, and defective actions of C_2_H_2_-ZNFs develop characteristic morphological and/or functional abnormalities depending on their actions, expressed timing and residing cells.

Although there are substantial numbers of exceptions, defective poly-ZNFs (e.g., BLI3, ZEBs, and ZICs) tend to be associated with the NDDs with gross abnormality in brain morphology and/or structure, whereas dysfunction of the C_2_H_2_-ZNFs harboring additional domains, such as KRAB and SCAN (e.g., ZNF18, ZNF34, ZNF81, ZNF427, ZNF673, ZNF804A, and ZBTB20) are linked to the development of NDDs with abnormality in higher-order brain functions, such as cognitive deficit, memory loss, and emotional changes, represented by ID, ASDs, schizophrenia, MDD, and/or BAD. C_2_H_2_-ZNFs are found throughout the organisms from yeasts to humans, whereas their numbers have exponentially expanded following the species evolution, particularly in vertebrates including humans (33). Poly-ZNFs tend to present from lower to higher organisms and mediate the fundamental functions shared by most of them, such as embryonic/fetal development, organogenesis, and limb formation (21). On the other hand, KRAB-ZNFs and SCAN-ZNFs, which appeared in the animal kingdom from vertebrates and mammals, respectively, show their numbers have significantly expanded in higher organisms, with the former demonstrating this trend more obviously (33). It is likely that the addition of these domains to C_2_H_2_-ZNFs, particularly the KRAB domain, appears to be required for supporting the functions specific to higher organisms, for example, sophisticated cognitive functions unique to humans (21). These pieces of evolutionary evidence on C_2_H_2_-ZNFs may explain our successful identification of specific C_2_H_2_-ZNF subtypes in particular forms of NDDs. For example, we found high accumulation of KRAB-ZNFs in ID, ASDs, and psychotic diseases that are associated with dysfunctions in higher-order brain functions, whereas defective poly-ZNFs appears to be linked to gross morphological brain abnormalities, including microcephaly, lissencephaly, and local hypoplasia/anomaly. This is also consistent with the previous finding that the characteristic expression of KRAB-ZNFs in the human brain compared to other primates including chimpanzees appears to be required for driving human-specific brain functions (16).

About two thirds of the KRAB-ZNF proteins are reported to bind retrotransposon sequences incorporated in the genome DNA, and act as protecting agents against reactivation and subsequent genome migration of these mobile elements (167). Retrotransposons cause various genetic diseases with their property of genome mutagenesis and chromosomal rearrangement (168). On the other hand, they are major driving forces for species evolution, participating in the development of a sophisticated gene regulatory network characteristic found in higher organisms by providing new regulatory elements and/or TF-binding sites through insertion of their long terminal repeat promoters (169). Thus, dense involvement of KRAB-ZNFs in neurobiology and neurogenesis might have been established in part through insertion of the regulatory elements originated from retrotransposons that harbor binding sites for KRAB-ZNFs into relevant key genes. Because retrotransposons facilitate the development of non-inherited gene regulatory diversity in brain neurons through their genome migration and subsequent mutagenic property in these non-dividing cells (170, 171), it is possible that dysfunction of the KRAB-ZNFs might influence this unique process mediated by retrotransposons by impacting their reactivation and further increase phenotypic variation of the NDD patients.

In conclusion, we performed the literature-based analysis on the roles of C_2_H_2_-ZNFs in brain development and pathogenesis of NDDs. We found that numerous C_2_H_2_-ZNFs play important roles in these physiologic and pathologic processes. We hope that this literature assessment will encourage the researchers' focus on C_2_H_2_-ZNFs in helping us extend our understanding of brain physiology and pathophysiology.

## Author Contributions

NA-N wrote the manuscript draft. RM created the table and edited the text. TK edited and created the final manuscript.

### Conflict of Interest

The authors declare that the research was conducted in the absence of any commercial or financial relationships that could be construed as a potential conflict of interest.
